# Synthetic receptor scaffolds significantly affect the efficiency of cell fate signals

**DOI:** 10.1038/s41598-024-56612-2

**Published:** 2024-03-09

**Authors:** Kirato Umene, Masahiro Kawahara

**Affiliations:** 1 Department of Chemistry and Biotechnology, Graduate School of Engineering, The University of Tokyo, 7-3-1 Hongo, Bunkyo-Ku, Tokyo, 113-8656 Japan; 2 Laboratory of Cell Vaccine, Microbial Research Center for Health and Medicine (MRCHM), National Institutes of Biomedical Innovation, Health and Nutrition (NIBIOHN), 7-6-8 Saito-Asagi, Ibaraki-Shi, Osaka, 567-0085 Japan

**Keywords:** Biotechnology, Molecular engineering, Protein design, Synthetic biology, Genetic engineering

## Abstract

Mimicry of receptor functions by designing synthetic receptors would be one of the recently hot research trends in cell engineering. While several types of synthetic receptors have been designed to induce desired cell fates in response to external stimuli, little is known about which receptor type signals more efficiently for inducing a certain cell fate. In this study, we compared the performance of three types of synthetic receptor scaffolds, *i.e.* myristoylated, cytosolic, and transmembrane types that signal through JAK-dependent phosphorylation of tyrosine motifs to transduce growth signaling. As a result, the phosphorylation levels of JAK and subsequent downstream signaling molecules were significantly maintained in the cytosolic type receptors, leading to more efficient cell growth than the other types. In contrast, the phosphorylation levels of JAK decreased in a motif-dependent manner in the transmembrane type receptors. Although various studies on receptor engineering based on domain or motif engineering have been reported, to our knowledge this study is the first to demonstrate that synthetic receptor scaffolds significantly affect the efficiency of cell fate signals. These findings are important for both receptor biology and receptor engineering, providing guidelines for rationally designing synthetic receptors that can transduce as efficient signaling as possible.

## Introduction

Cells respond to changes in their extracellular environments through receptors expressed on the plasma membrane or in the cytosol. Hormones and cytokines activate their specific receptors and trigger intracellular signal transduction, which dynamically changes post-translational modifications of various intracellular molecules to induce cell fates such as proliferation, differentiation, migration, death, and even a variety of immune reactions for host defense. One of the recently hot research trends in cell engineering would be mimicry of receptor functions by designing synthetic receptors to enable cells to exert desired functions in response to external stimuli^1,2^. Synthetic notch (synNotch) receptors realize intercellular communications by arbitrarily coupling transcriptional regulations with external antigen ligands^3,4^. When expressed on T cells, chimeric antigen receptors (CAR) recognize cancer-specific antigens on the plasma membrane and induce tumor-killing activities through the CD3ζ-based signaling domains^5,6^.

Thus, it would theoretically be possible to induce desired cell fates in response to any external stimuli with rational design of synthetic receptors. A major aspect of the rational design is how we engineer the signaling domain of synthetic receptors on a domain or motif basis to regulate signaling properties^1,2^. Another important aspect would be how we choose an appropriate receptor scaffold among those with different structural characteristics. Such synthetic receptor scaffolds could be categorized into two types according to cell membrane permeability of the ligands. One type receives soluble or transmembrane proteins as an extracellular ligand and thus generally spans the plasma membrane. The marked examples are synNotch and CAR, which we have introduced in the previous paragraph. Particularly, CAR-T cells attained high therapeutic effects on CD19-positive B cell lymphoma and have already reached clinical applications^6^. In addition, we previously developed a variety of cytokine receptor-based cell fate-inducing CARs (cfiCARs) that induce various cell fates including proliferation, differentiation, migration, and apoptosis in response to soluble antigens, depending on the combination of signaling domains and host cells^7–14^. The other type of the synthetic receptor scaffolds receives membrane-permeable small molecules through a dimerization domain and thus is expressed intracellularly in the cytosol or at the inner leaflet of the plasma membrane with lipid modification. A representative example is a chemical inducer of dimerization (CID) system using an F36V mutant of FK506-binding protein 12 (FKBP_F36V_), which can control the activation of not only receptors but also various signal transducers and transcription factors in response to a synthetic dimerizer AP1903 or AP20187^15,16^. Particularly, iCasp9, an inducible suicide gene, has progressed to Phase I clinical trials as a safety switch in cell therapy^17^. In addition, we previously developed designer receptors that can activate target signaling molecules by appropriately arranging domains and tyrosine motifs^18–20^. Such a designer receptor was also utilized for phenotypic screening of tyrosine motifs that efficiently induce cell proliferation^21^.

The above-mentioned transmembrane and intracellular receptor types have their own advantages. The transmembrane type has more choices of ligands, and the membrane raft facilitates association between the receptor chains. On the contrary, the intracellular type has a higher degree of conformational freedom in the dimer/oligomer structure after ligand binding^22^. However, little is known about which receptor type signals more efficiently for inducing a certain cell fate. This is due to the lack of studies that compare the signaling properties of the different receptor types in parallel. Therefore, in this study, we aim to obtain useful knowledge on design strategies for synthetic receptors by systematically comparing the signaling intensity of the different receptor types. To accomplish this, we employ a series of signaling domains with various tyrosine motifs and test the activity of cell growth, which is the most fundamental cell fate.

## Results

### Designing three types of the synthetic receptor scaffolds

**Figure 1:**
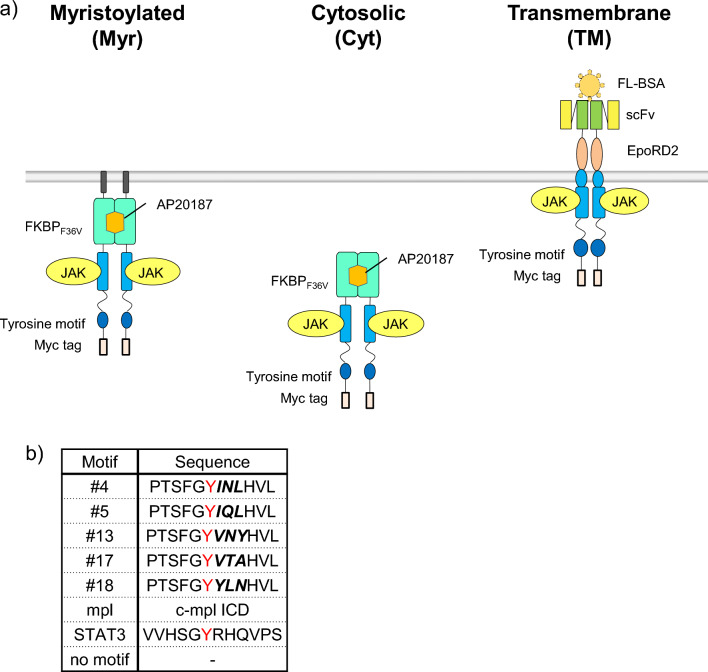
Outline of the chimeric receptor constructs. (**a**) Illustration of the three-type chimeric receptors. The myristoylated (Myr) and cytoplasmic (Cyt) receptors are activated by a cell-permeable synthetic dimerizer AP20187, whereas the transmembrane (TM) receptor is activated by fluorescein-conjugated BSA (FL-BSA). (**b**) The amino acid sequence of the tyrosine motifs incorporated in the chimeric receptors. The clones (#4, 5, 13, 17, 18) are derived from a three amino acid-randomized library of the STAT1-binding motif. The randomized positions are indicated as bold and italic. The tyrosine residues are colored in red.

We aimed to systematically compare the signaling properties of the following three types of the synthetic receptor scaffolds (Fig. 1a). A myristoylated (Myr) type encodes an FKBP_F36V_-based receptor scaffold and is localized at the inner leaflet of the plasma membrane via an N-terminal myristoylation signal sequence. A cytosolic (Cyt) type also encodes the FKBP_F36V_-based scaffold and is simply expressed in the cytosol without any signal sequences. The Myr and Cyt types are commonly activated by a membrane-permeable dimerizer ligand AP20187. A transmembrane (TM) type encodes anti-fluorescein single-chain Fv and the D2 domain of erythropoietin receptor (EpoRD2) in the extracellular domain and is activated by an oligomeric antigen fluorescein-conjugated BSA (FL-BSA). These three types commonly encode the JAK-binding domain of c-mpl and a tyrosine motif as a signal-transducing domain and a Myc tag at the C-terminus. The tyrosine motif sequences include cell growth-inducing motif sequences selected from a library based on the STAT1-binding motif (#4, #5, #13, #17, #18)^21^, the intracellular domain of c-mpl, the STAT3-binding motif, and no motif as a negative control (Fig. 1b).

### Cyt receptor transduces the most effective growth signaling

**Figure 2:**
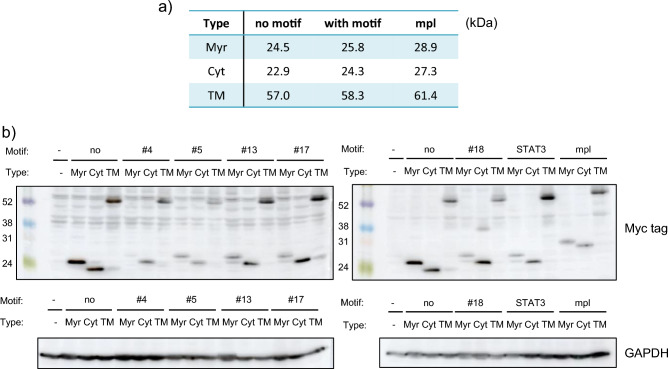
Confirmation of the receptor expression. (**a**) The estimated molecular mass of each chimeric receptor. (**b**) Western blotting to detect expression of the chimeric receptors. Blots with Myc tag and GAPDH represent receptor expression levels and loading controls, respectively. Uncropped blot images are provided in Supplementary Information (Supplementary Fig. 3).

The receptor constructs were genetically introduced into murine IL-3-dependent Ba/F3 cells, in which we easily compare the properties of growth signaling induced by the synthetic receptors. First, we confirmed the expression of the synthetic receptors in western blot analysis. As a result, all of the synthetic receptors were expressed with the expected molecular mass (Fig. 2). The expression levels differed among the receptors, but not depending on the types of the receptor scaffolds.

**Figure 3:**
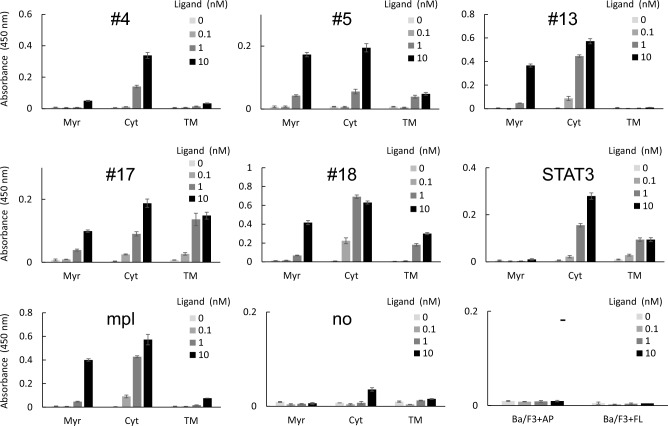
Growth assay. Cells were cultured with the indicated concentrations of the ligand AP20187 (for the Myr and Cyt receptors) or FL-BSA (for the TM receptors) for 3 days with the initial cell density of 7.0 × 10^3^ cells/mL. Cell growth levels were measured by a colorimetric assay. The (-) panel indicates the data of parental Ba/F3 cells cultured with each ligand (AP20187 or FL-BSA), which means the background levels. The data are represented as mean ± SE (n = 3, independent experiments).

Next, we performed a cell growth assay to examine whether the cells expressing the synthetic receptors could induce growth signals in response to the corresponding ligand AP20187 (for the Myr and Cyt types) or FL-BSA (for the TM type). The results showed that in all motif sequences the Cyt type receptors showed higher cell growth levels than the TM type receptors (Fig. 3). When compared with the corresponding Cyt type receptors, TM #13 was non-responsive, TM #4, #5, and mpl were slightly responsive, TM #18 and STAT3 were moderately responsive, and TM #17 was highly responsive and thus comparable to Cyt #17. The Cyt type showed higher growth activity than the Myr type, indicating that membrane localization is not required for growth signaling of the synthetic receptors tested in this study.

### Signaling analysis revealed the dramatic difference of signaling intensities between Cyt and TM receptors

**Figure 4:**
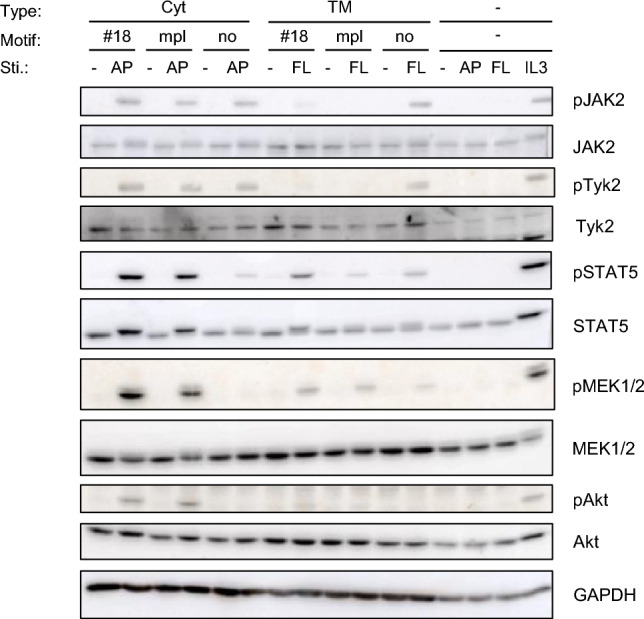
Exploring the ligand-dependent activation levels of signaling molecules. Cells were depleted in the absence of IL-3 and stimulated for 15 min without any factors (-) or with 10 nM AP20187 (AP), 10 nM FL-BSA (FL), or 1 ng/mL IL-3 (IL3). The cell lysates were subjected to SDS-PAGE and western blotting. Blots with the antibodies recognizing each whole molecule and GAPDH serve as loading controls. The most right four lanes in which the type and motif are (-) represent parental Ba/F3 cells as stimulation controls. Uncropped blot images are provided in Supplementary Information (Supplementary Fig. 4).

To explore the reason why the Cyt type receptors transduce stronger growth signals than the TM type receptors, we performed signaling analysis to examine the phosphorylation of signaling molecules in western blotting (Fig. 4). We chose #18 as the highest growth-inducing tyrosine motif clone, mpl as the natural receptor-derived tyrosine motifs, and no motif as a negative control. Consequently, JAK2 and Tyk2, which are the most upstream tyrosine kinases activated by receptor dimerization, exhibited interesting phosphorylation properties. Intriguingly, the cytosolic type showed similar phosphorylation levels of JAK2 and Tyk2 regardless of the presence or absence of the motifs, while the TM type showed the phosphorylation levels of JAK2 and Tyk2 decreased in the presence of the tyrosine motifs. Furthermore, the Cyt type induced higher levels of phosphorylation of downstream growth signaling molecules than the TM type.

**Figure 5:**
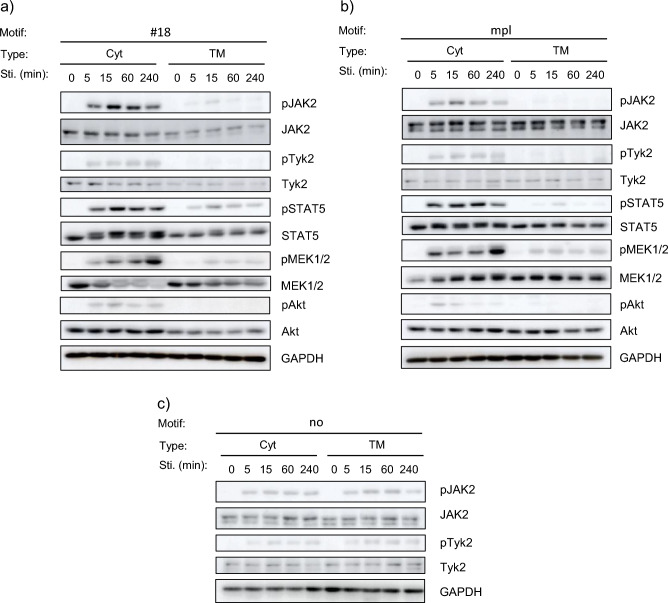
Comparing the time-dependent signaling levels between the Cyt and TM receptors. Cells were depleted in the absence of IL-3 and stimulated for the indicated time periods with 10 nM AP20187 (for the Cyt receptors) or 10 nM FL-BSA (for the TM receptors). The cell lysates were subjected to SDS-PAGE and western blotting. Blots with the antibodies recognizing each whole molecule and GAPDH serve as loading controls. The receptor constructs incorporating (**a**) motif #18, (**b**) the intracellular domain of c-mpl, and (**c**) no motif were analyzed. Uncropped blot images are provided in Supplementary Information (Supplementary Figs. 5, 6, and 7).

In order to reliably demonstrate that the Cyt type signals stronger than the TM type, we compared the Cyt type and TM type in parallel for #18 and mpl by varying the stimulation time (0, 5, 15, 60, or 240 min) with each ligand. Consequently, the phosphorylation levels of all signaling molecules tested (JAK2, Tyk2, STAT5, MEK and Akt) in the Cyt type were much higher than those in the TM type for all stimulation time periods (Fig. 5a,b). While the phosphorylation levels of STAT5 and Akt decreased in a time-dependent manner, the phosphorylation levels of MEK lasted long and reached the maximum at 240 min in the Cyt type. On the other hand, for no motif, JAK2 and Tyk2 were phosphorylated in similar levels for both Cyt and TM types (Fig. 5c).

Thus, the TM type led to motif-dependent decrease of activation levels of the most upstream kinases JAK2 and Tyk2, but the Cyt type did not. This phenomenon was also manifested in the phosphorylation levels of downstream signaling molecules. Consequently, the Cyt type activated signaling with higher intensity and ultimately promoted faster cell growth.

## Discussion

In this study, we compared the performance of three types of synthetic receptor scaffolds, *i.e.* myristoylated (Myr), cytosolic (Cyt), and transmembrane (TM) types that signal through JAK-dependent phosphorylation of tyrosine motifs. As a result, the phosphorylation levels of JAK and subsequent downstream signaling molecules were significantly maintained in the Cyt type receptors, leading to more efficient cell growth than the other types. In contrast, the phosphorylation levels of JAK decreased in a motif-dependent manner in the TM type receptors. Although various studies on receptor engineering based on domain or motif engineering have been reported, to our knowledge this study is the first to demonstrate that synthetic receptor scaffolds significantly affect the efficiency of cell fate signals. This finding would be interesting in both receptor biology and receptor engineering aspects.

The TM type receptors are present in various organelles such as ER, Golgi, and endosomes in the cytoplasm, whereas the ligand (FL-BSA) only acts those localized on the plasma membrane. Therefore, it is intrinsically difficult to precisely adjust the number of dimerized receptors among Cyt, Myr, and TM type receptors. Thus, we simply expressed these receptors in the same way using the same expression vector. Consequently, the Cyt type and the TM type receptors with no motif exhibited similar phosphorylation levels of JAK (Fig. 5c), suggesting that the TM type receptors were not in extremely unfavorable conditions with respect to dimerization. Under this condition, the TM type receptors with the motifs (#18 and mpl) resulted in much weaker phosphorylation levels of downstream signaling molecules than the corresponding Cyt type receptors, which we believe is a fair evaluation.

It has been reported that the Myr type receptors are not completely localized at the membrane, but is in equilibrium between the cytoplasmic fraction (corresponding to the Cyt type) and the membrane-localized fraction^23^. For example, Myr #13 induced a certain degree of proliferation probably due to the effects of the cytoplasmic fraction (Fig. 3). Therefore, in this paper we mainly discussed the differences in signaling intensities between Cyt and TM types in order to avoid complicated discussions due to ambiguity of the Myr type.

In the signaling analyses, the JAK activation levels decreased tyrosine motif-dependently in the TM type receptors, whereas this phenomenon did not occur in the Cyt type receptors. The results suggest that signaling molecules with tyrosine motif-binding SH2 domains are involved in growth suppression in the TM type receptors. Furthermore, as clearly shown in Fig. 5, the phosphorylation levels of downstream signaling molecules were weaker overall in the TM type, which was correlated with the phosphorylation levels of JAK. Taken together, we could hypothesize that a molecule that has an SH2 domain, can inactivate JAK, and works efficiently on the plasma membrane would be involved in the signaling events of the TM type receptors.

Considering the first two conditions of the above hypothesis, suppressor of cytokine signaling (SOCS) and SH2 domain-containing phosphatases (SHP1 and SHP2) would be candidate signaling molecules that have SH2 domains and can inactivate JAK^24^. Among these, while SOCS and SHP2 have not been reported to be localized at the plasma membrane, the tyrosine phosphatase SHP1 has been reported to have a lipid raft-binding motif^25–27^, suggesting that SHP1 may be membrane-localized and predominantly act on the TM type receptors.

As a negative regulator, SHP1 preferentially binds to the immunoreceptor tyrosine-based inhibitory motif (ITIM) with a consensus sequence (I/V/L)xYxx(L/V), when the tyrosine residue of the motif is phosphorylated^28^. When ITIM is present in the signaling domain of receptors, SHP1 dephosphorylates kinases associated with the receptors. Interestingly, the results of the growth assay (Fig. 3) revealed that #13, whose cell growth was extremely suppressed in the TM type, has another tyrosine residue at the Y + 3 position, which generates the completely matched ITIM sequence (VNYHVL). In addition, #4 and #5, whose cell growth was strongly suppressed in the TM type, have YxxL, which matches the latter half of the ITIM sequence (#4: FGYINL, #5: FGYIQL). In addition, the intracellular domain of the natural receptor mpl, whose cell growth was strongly suppressed in the TM type at similar levels to #4 and #5, have two YxxL motifs out of the three motifs (MDYRRL, HSYLPL, LSYWQQ). Of note, the cell growth level of #17 (FGYVTA) was not suppressed in the TM type compared to the Cyt type, which implies that SHP1 may not preferentially bind to the YxxA motif. Since #18 (FGYYLNH) and the STAT3-binding motif (SGYRHQ) have intermediate cell growth levels in the TM type, SHP1 may bind to these motifs at moderate levels.

As the name indicates, ITIM has been considered to be suppressive for receptor signaling. However, our results of #13 suggest that ITIM lost the suppressive functions when located in the cytosol. This is an interesting finding and also importantly suggests that receptors are susceptible to negative regulation by tyrosine phosphatases at the vicinity of the plasma membrane rafts but are released from the negative regulation in the cytosol. Therefore, choosing the Cyt type rather than the TM type would be reasonable to create synthetic receptors with more intensive phosphorylation levels and eventually higher signaling efficiency. Although we have demonstrated the importance of the receptor type for inducing growth of Ba/F3 cells in the present study, this knowledge could also be applied to other cells and cell fates, by which synthetic receptors could be recognized as an increasingly important and promising tool for cell fate regulation with high efficiency.

## Materials and methods

### Plasmid construction

The chimeric receptor constructs were subcloned into a retroviral transfer plasmid pMK-stuffer-IPTG as described previously^21^. Owing to the internal ribosomal entry site (I) –puromycin resistance (P) gene cassette, retrovirally transduced cells stably expressing the chimeric receptors can be selected by culturing cells with puromycin. We conducted plasmid construction and sequencing according to standard protocols. The amino acid sequences of the myristoylated, cytosolic, and transmembrane type chimeric receptors are shown in Supplementary Information (Supplementary Fig. 1).

### Retroviral transduction

Ba/F3 cells (RIKEN Cell Bank #RCB0805, Ibaraki, Japan) were retrovirally transduced as described previously^21^. Briefly, retroviral packaging cells were transiently transfected with each of the constructed retroviral transfer plasmids, and the retroviral supernatant was used for transducing Ba/F3 cells on a Retronectin (TakaraBio)-coated plate. The transduced cells were selected in the presence of 1 µg/mL puromycin.

### Cell growth assay

Cell growth assay was also performed as described previously^21^. Briefly, cells cultured with 1 ng/mL of IL-3 (ThermoFisher Scientific) were washed twice with PBS and inoculated into 24-well plates with the indicated concentrations of the ligand AP20187 (TakaraBio) or FL-BSA (Sigma-Aldrich). Viable cell densities were estimated using Cell Counting Kit-8 (Dojindo Laboratories) by measuring absorbance at 450 nm using GloMax Discover Microplate Reader (Promega).

### Western blotting

Western blotting was also performed as described previously^21^. For confirming receptor expression, cells (1.0 × 10^6^ cells) cultured with 1 ng/mL of IL-3 were simply harvested. For the signaling analysis, cells cultured with 1 ng/mL of IL-3 were washed twice with PBS and then cultured without IL-3 for 10–13 h. The depleted cells (1.0 × 10^6^ cells) were stimulated with 10 nM AP20187, 10 nM FL-BSA (FL), or 1 ng/mL IL-3 at 37˚C for the indicated time periods and then harvested. The stimulated cells were treated with 2 mM Na_3_VO_4_ in ice-cold PBS to inhibit dephosphorylation. Subsequently, the cells were lysed with 100 μl of lysis buffer containing 1 mM Na_3_VO_4_, incubated on ice for 10 min, and centrifuged at 22,300 g for 10 min at 4 ℃. The supernatant was mixed with 33 μl of 4X SDS-PAGE Sample Buffer (Tokyo Chemical Industry) and boiled at 98 ℃ for 5 min. The lysates were subjected to the SDS-PAGE/western blot analysis. The antibodies used in western blotting are listed in Supplementary Information (Supplementary Fig. 2).

### Supplementary Information

Supplementary Information.

## Data Availability

All data generated or analyzed during this study are included in this published article and its Supplementary Information.
