# Supplementary material for: Synthetic receptor scaffolds significantly affect the efficiency of cell fate signals
Source: Sci Rep. 2024 Mar 9;14:5801. doi: 10.1038/s41598-024-56612-2 (PMC10925030; doi:10.1038/s41598-024-56612-2)
Supplement: Supplementary file 1 — Supplementary Information. [file 41598_2024_56612_MOESM1_ESM.pdf]

Supplementary Information

Synthetic receptor scaffolds significantly affect the efficiency of cell fate signals

Kirato Umene[1], Masahiro Kawahara[1,2,*]

[1]Department of Chemistry and Biotechnology, Graduate School of Engineering, The University of Tokyo, 7-3-1 Hongo, Bunkyo-ku, Tokyo 113-8656, Japan.

[2]Laboratory of Cell Vaccine, Microbial Research Center for Health and Medicine (MRCHM), National Institutes of Biomedical Innovation, Health and Nutrition (NIBIOHN), 7-6-8 Saito-Asagi, Ibaraki-shi, Osaka 567-0085, Japan.

[*]Correspondence: Masahiro Kawahara, Laboratory of Cell Vaccine, Microbial Research Center for Health and Medicine (MRCHM), National Institutes of Biomedical Innovation, Health and Nutrition (NIBIOHN), 7-6-8 Saito-Asagi, Ibaraki-shi, Osaka 567-0085, Japan.
E-mail: m-kawahara@nibiohn.go.jp

a) Myristoylated type (Myr)

MGSSKSKPKDPSQRGSGGVQVETISPGDGRTFPKRGQTCVVHYTGMLEDGKKVDSS
RDRNKPFKFMLGKQEVIRGWEEGVAQMSVGQRAKLTISPDYAYGATGHPGIIPPHATL
VFDVELLKLEGSGRWQFPAHYRRLRHALWPSLPDLHRVLGQYLRDTAALSPPKATVS
DTCEEVEPSLLEILPKSSERTPLPLRVGGGGSGGGGSGGGGSRVmotif_sequenceIDEQK
LISEEDL

b) Cytosolic type (Cyt)

MGVQVETISPGDGRTFPKRGQTCVVHYTGMLEDGKKVDSSRDRNKPFKFMLGKQEV
IRGWEEGVAQMSVGQRAKLTISPDYAYGATGHPGIIPPHATLVFDVELLKLEGSGRWQ
FPAHYRRLRHALWPSLPDLHRVLGQYLRDTAALSPPKATVSDTCEEVEPSLLEILPKSS
ERTPLPLRVGGGGSGGGGSGGGGSRVmotif_sequenceIDEQKLISEEDL

c) Transmembrane type (TM)

METDTLLLWVLLLWVPGSTGDYPYDVPDYAGAQPADVVMTQTPLSLPVSLGDQASIS
CRSSQSLVHSNGNTYLRWYLQKPGQSPKVLIYKVSNRVSGVPDRFSGSGSGTDFTLKI
NRVEAEDLGVYFCSQSTHVPWTFGGGTKLEIKSSADDAKKDAAKKDDAKKDDAKK
DGGVKLDETGGGLVQPGGAMKLSCVTSGFTFGHYWMNWVRQSPEKGLEWVAQFR
NKPYNYETYYSDSVKGRFTISRDDSKSSVYLQMNNLRVEDTGIYYCTGASYGMEYL
GQGTSVTVSGSGVLLDAPVGLVARLADESGHVVLRWLPPPETPMTSHIRYEVDVSAG
NGAGSVQRVEILEGRTECVLSNLRGRTRYTFAVRARMAEPSFGGFWSAWSEPVSLLTP
SDLDPDIISLVTALHLVLGLSAVLGLLLLRWQFPAHYRRLRHALWPSLPDLHRVLGQY
LRDTAALSPPKATVSDTCEEVEPSLLEILPKSSERTPLPLRVGGGGSGGGGSGGGGSRV
motif_sequenceIDEQKLISEEDL

d) c-mpl ICD

RWQFPAHYRRLRHALWPSLPDLHRVLGQYLRDTAALSPPKATVSDTCEEVEPSLLEIL
PKSSERTPLPLCSSQAQMDYRRLQPSCLGTMPLSVCPPMAESGSCCTTHIANHSYLPLS
YWQQP

Supplementary Figure 1 The amino acid sequences of the myristoylated, cytosolic, and transmembrane type chimeras.

(a-c) Each motif sequence in Fig. 1b was inserted into the motif_sequence region in each construct except c-mpl ICD.

(d) In the case of the c-mpl intracellular domain (ICD), the described sequence was substituted for the JAK-binding domain, the RV$(G_4S)_3$RV linker, and the motif_sequence region of each construct in (a-c).

Purple: myristoylation signal. Black: linker or extra sequence. Green: $FKBP_{F36V}$. Blue: truncated (the JAK-binding domain) or whole intracellular domain of c-mpl. Brown: Myc tag. Grey: leader sequence derived from immunoglobulin κ chain. Light green: HA tag. Yellow: FL-specific scFv clone 4M5.3. Orange: D2 domain of erythropoietin receptor. Light blue: transmembrane domain of c-mpl.

Antibody	Manufacturer	Cat#
rabbit anti-Myc tag	Bethyl Laboratories	A190-105A
rabbit anti-GAPDH	Cell Signaling Technology	5174
rabbit anti-phospho-JAK2 (Y1007/1008)	Cell Signaling Technology	3776
rabbit anti-JAK2	Santa Cruz Biotechnology	sc-294
rabbit anti-phospho-Tyk2 (Y1054/1055)	Cell Signaling Technology	9321
rabbit anti-Tyk2	Santa Cruz Biotechnology	sc-169
rabbit anti-phospho-STAT5 (Y694)	Cell Signaling Technology	9351
rabbit anti-STAT5	Santa Cruz Biotechnology	sc-835
rabbit anti-phospho-MEK1/2 (S217/221)	Cell Signaling Technology	9154
rabbit anti-MEK1/2	Cell Signaling Technology	8727
rabbit anti-phospho-Akt (T308)	Cell Signaling Technology	13038
rabbit anti-Akt	Cell Signaling Technology	9272
HRP-conjugated goat anti-rabbit IgG	Thermo Fisher Scientific	G-21234

Supplementary Figure 2 The antibodies used in Western blotting.

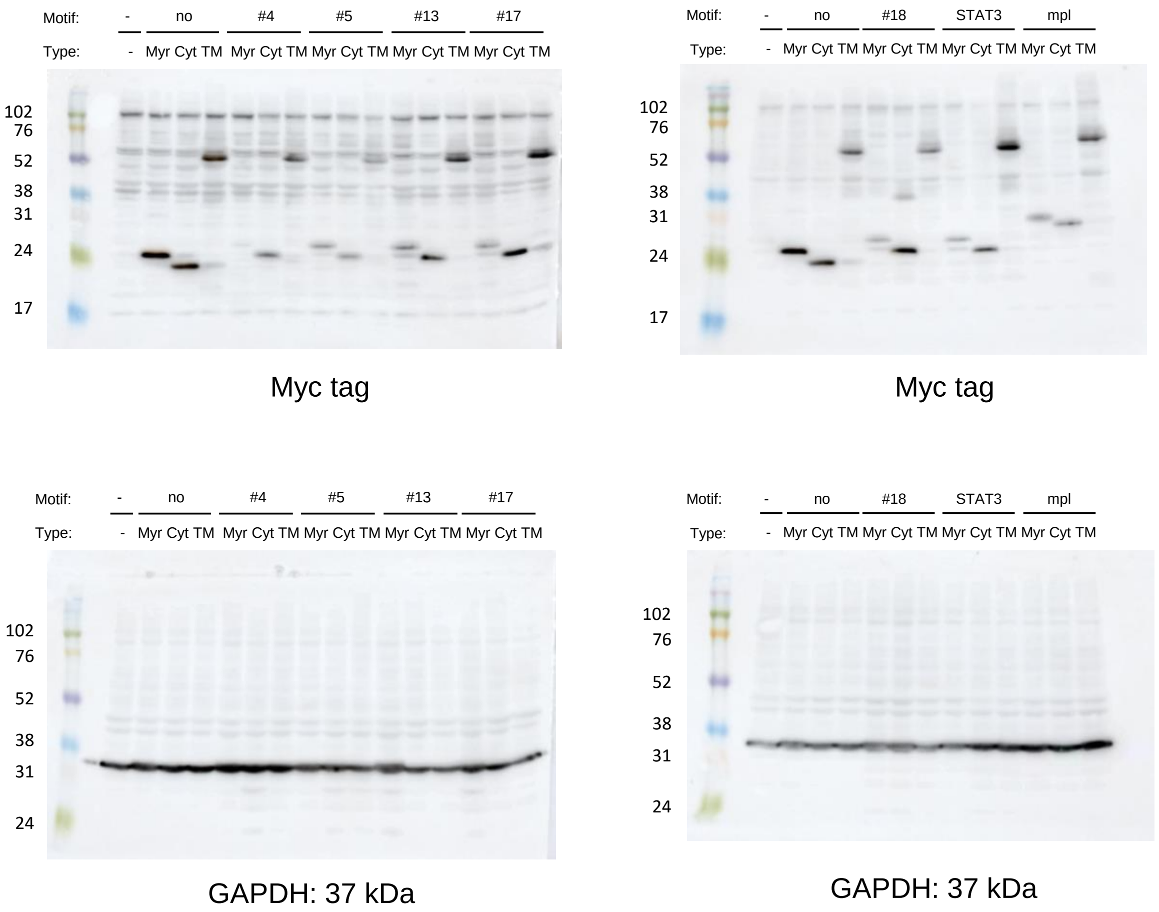

Supplementary Figure 3. Uncropped blot images for Fig. 2b.

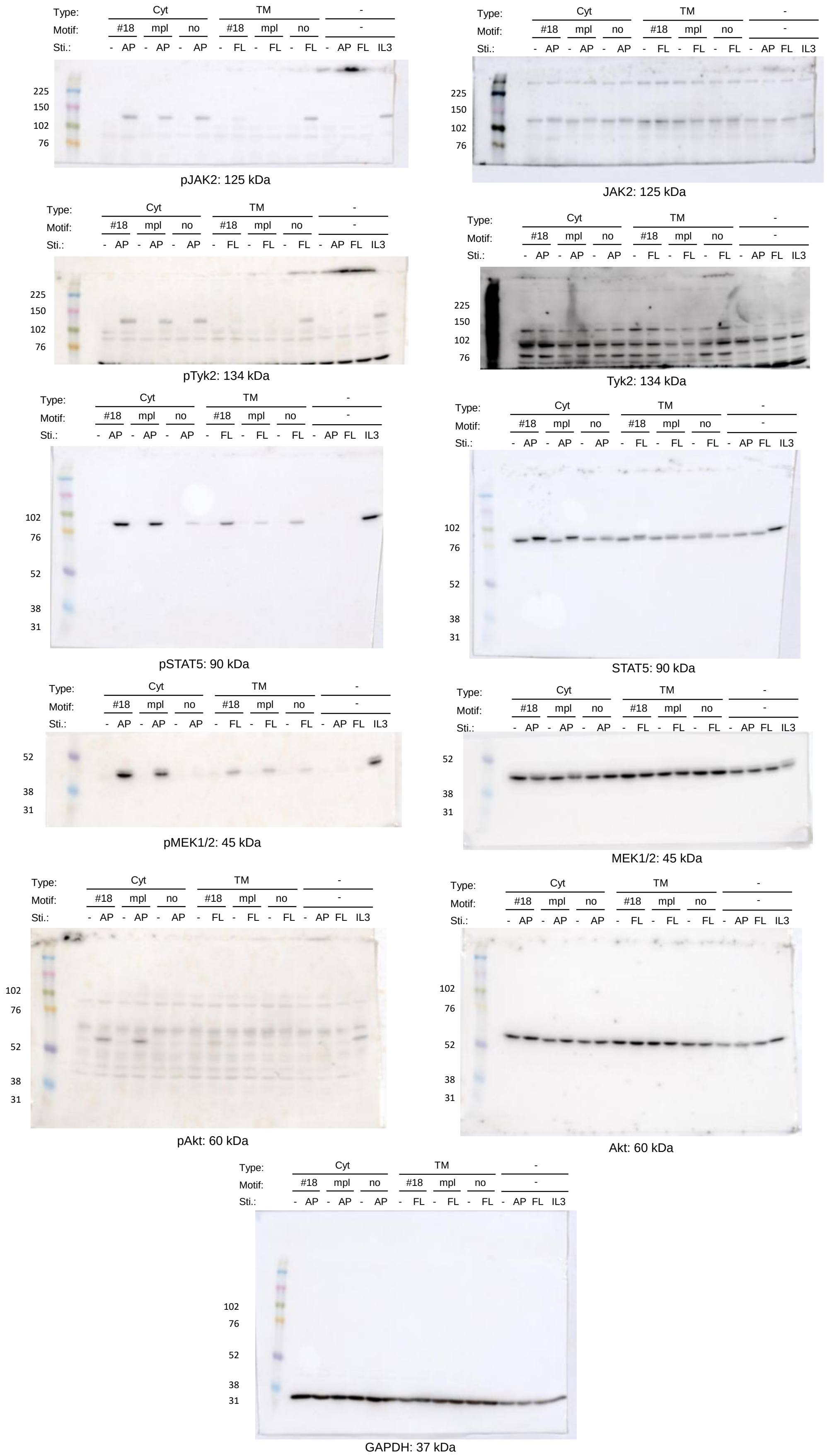

Supplementary Figure 4. Uncropped blot images for Fig. 4.

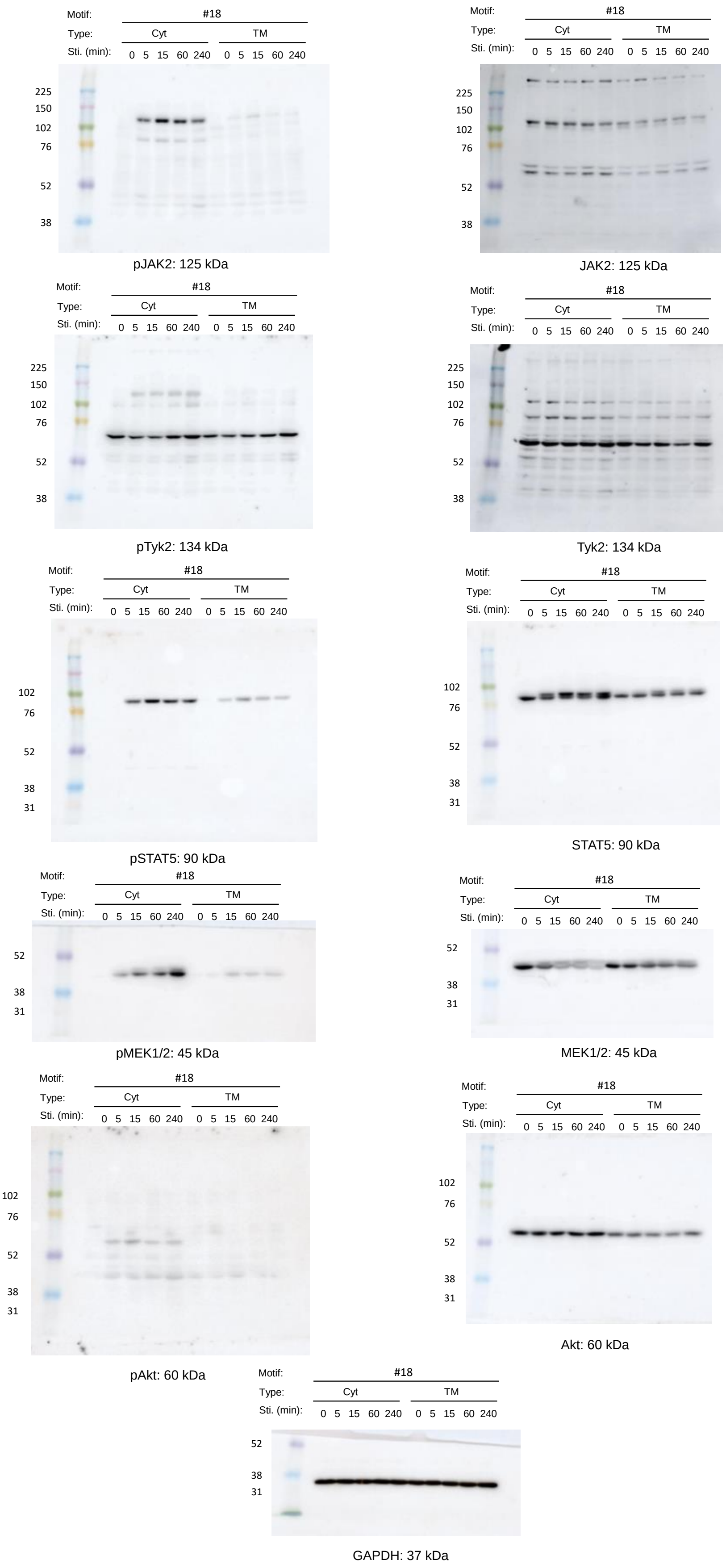

Supplementary Figure 5. Uncropped blot images for Fig. 5a.

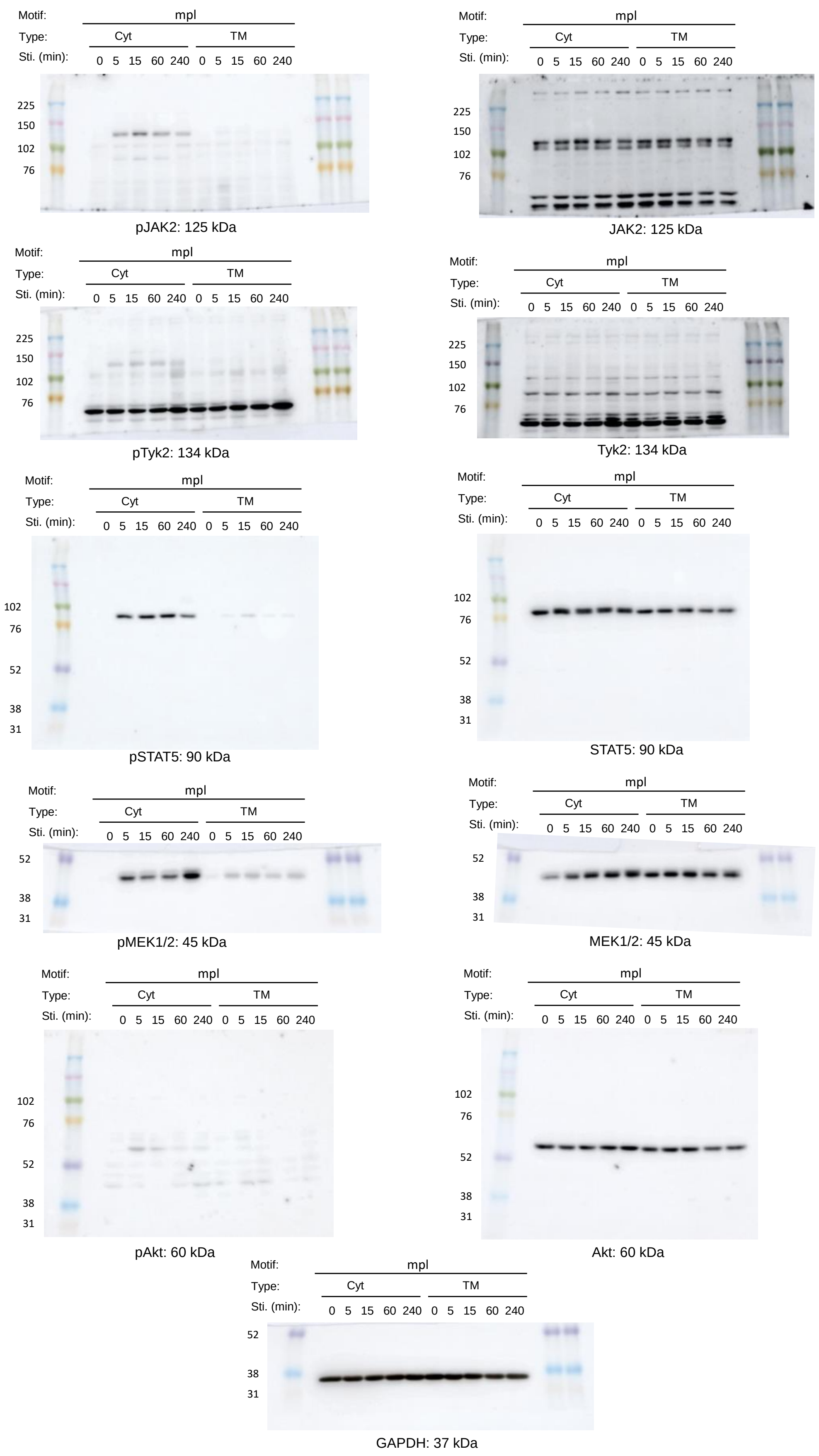

Supplementary Figure 6. Uncropped blot images for Fig. 5b.

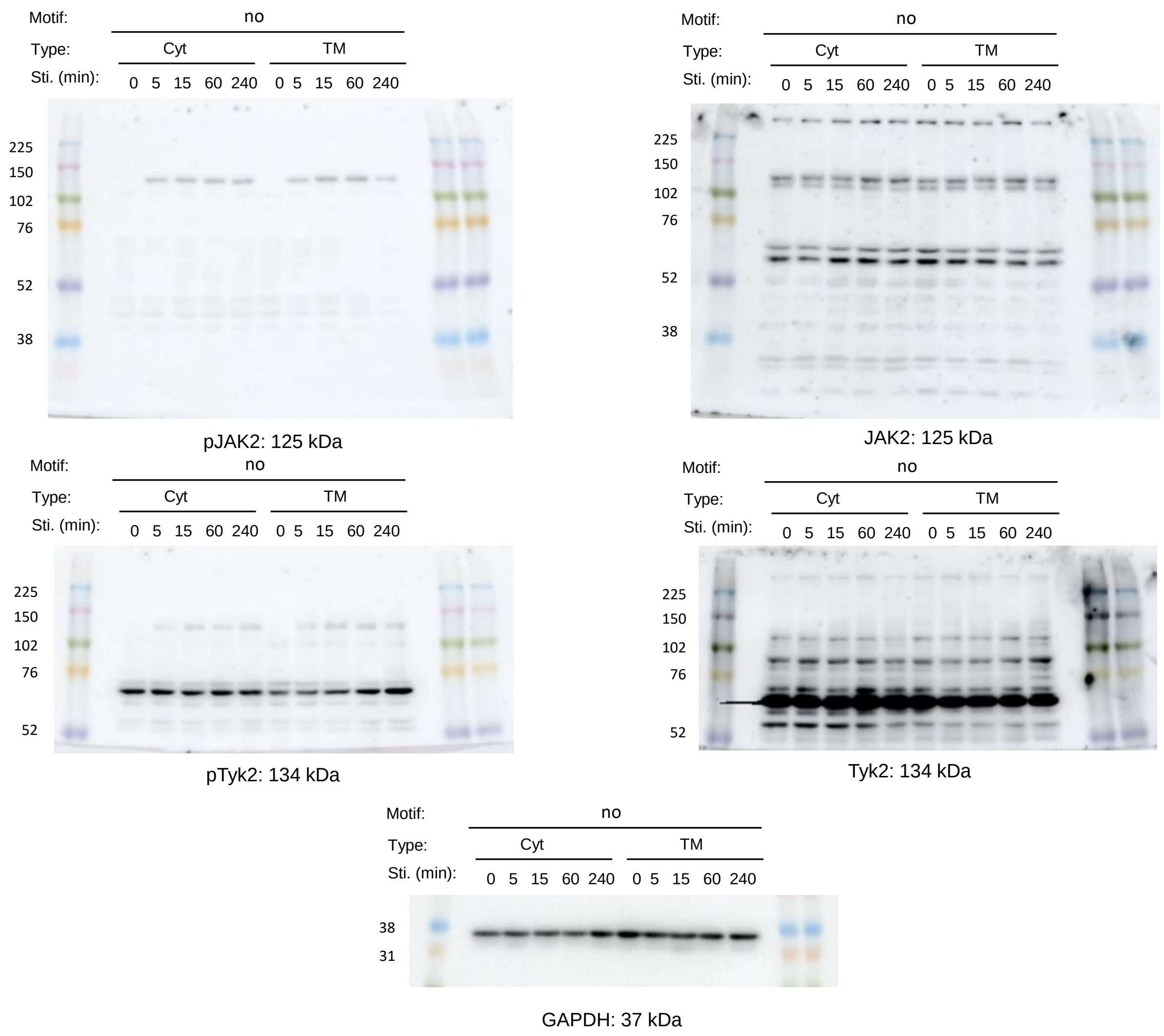

Supplementary Figure 7. Uncropped blot images for Fig. 5c.
